# ^87^Sr/^86^Sr age determination by rapidly formed spherical carbonate concretions

**DOI:** 10.1038/s41598-019-38593-9

**Published:** 2019-01-30

**Authors:** Hidekazu Yoshida, Yoshihiro Asahara, Koshi Yamamoto, Nagayoshi Katsuta, Masayo Minami, Richard Metcalfe

**Affiliations:** 1 Material Research Section, Nagoya University, University Museum, Chikusa, Nagoya Japan; 2 Graduate School of Environmental Studies, Nagoya University, Chikusa, Nagoya Japan; 3 Faculty of Education, Gifu University, Yanagido, Gifu Japan; 4 Institute for Space-Earth Environmental Research, Nagoya University, Chikusa, Nagoya Japan; 5 Quintessa UK, Newtown Road, Henley-on-Thames, Oxfordshire UK

**Keywords:** Geochemistry, Geochemistry

## Abstract

Isolated spherical carbonate concretions are frequently observed in finer grained marine sediments of widely varying geological age. Recent studies on various kinds of spherical carbonate (CaCO_3_) concretions revealed that they formed very rapidly under tightly constrained conditions. However, the formation ages of the isolated spherical carbonate concretions have never been determined. Here we use ^87^Sr/^86^Sr ratios to determine the ages of these spherical concretions. The studied concretions formed in the Yatsuo Group of Miocene age in central Japan. Some formed post-mortem around tusk-shells (*Fissidentalium* spp.), while other concretions have no shell fossils inside. The deformation of sedimentary layers around the concretions, combined with geochemical analyses, reveal that Sr was incorporated into the CaCO_3_ concretions during their rapid formation. Strontium isotopic stratigraphy using ^87^Sr/^86^Sr ratios of all concretions indicates an age of 17.02 ± 0.27 Ma, with higher accuracy than the ages estimated using micro-fossils from the Yatsuo Group. The results imply that the ^87^Sr/^86^Sr ratio of isolated spherical carbonate concretions can be applied generally to determine the numerical ages of marine sediments, when concretions formed soon after sedimentation. The ^87^Sr/^86^Sr age determinations have high accuracy, even in cases without any fossils evidence.

## Introduction

Spherical carbonate (CaCO_3_) concretions are observed widely throughout the world in marine sediments of varying geological ages. The concretions are typically enriched in Sr compared to the surrounding sedimentary rock matrices. The Ca and Sr in the concretions originate in marine water. Typically the concretions have sharp boundaries with the surrounding sedimentary rock matrices^1–4^. Over several decades there have been many mineralogical and geochemical studies of the concretion formation process during sediment burial and diagenesis^5–11^. However, the ^87^Sr/^86^Sr isotopic ratios have so far not been used to determine the ages of concretions.

**Figure 1:**
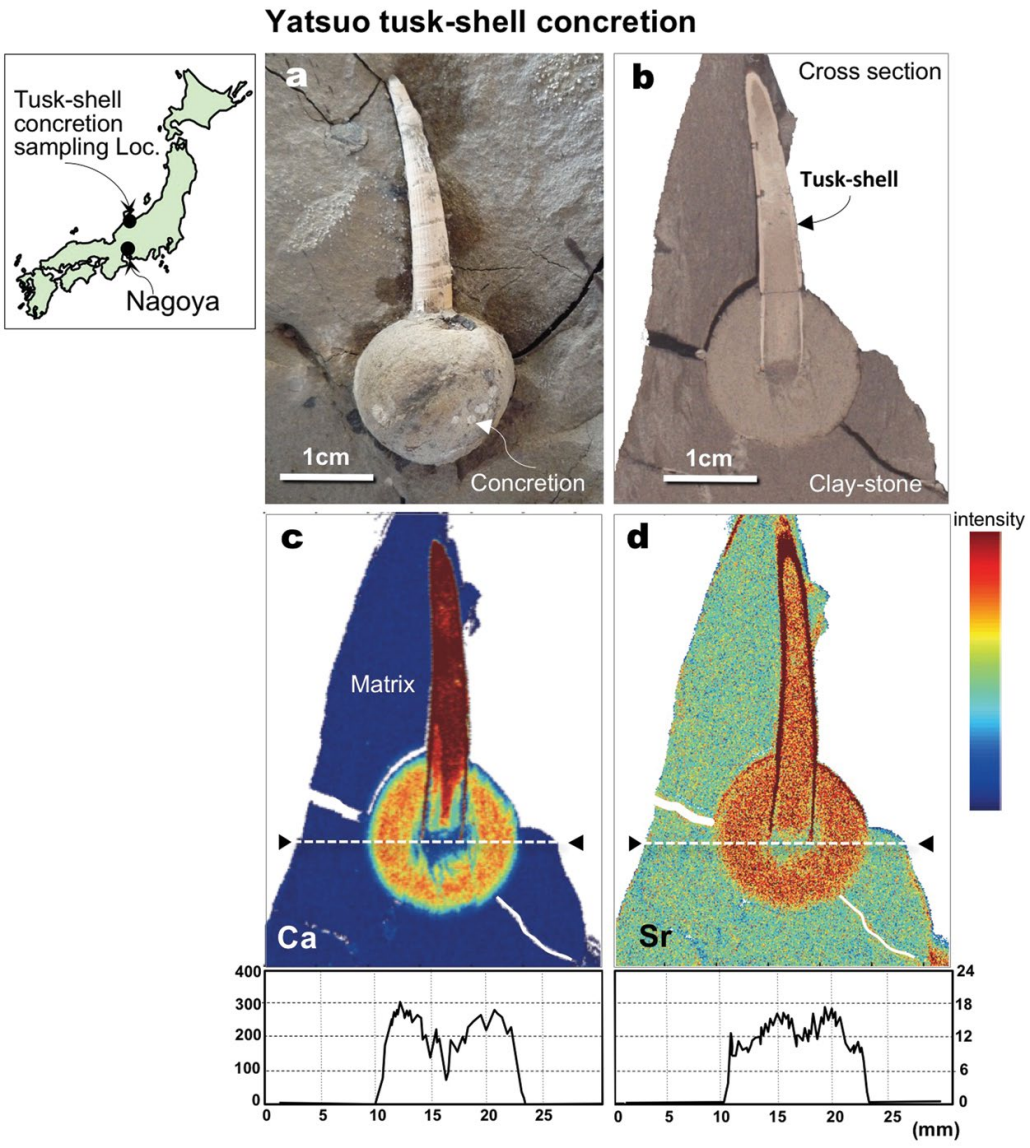
Ca and Sr distribution in and around the tusk-shell concretion. (**a**) Occurrence of a tusk-shell concretion formed around the mouth of a tusk-shell (*Fissidentalium* spp.) and, (**b**) a cross section through the tusk-shell showing the internal texture. (**c**,**d**) SXAM Ca and Sr X-ray intensity in and around the cut surface of a tusk-shell concretion. A sharp boundary between the concretion and matrix is also defined by the both elemental distribution. Index map is based on the data of Geospatial Information Authority of Japan website (http://www.gsi.go.jp/ENGLISH/index.html). Figures a–c are referred from Yoshida *et al*.^3^. All photographs (**a**,**b**) shown here are taken by H. Yoshida.

Yoshida *et al*.^4^ determined that generally, isolated spherical carbonate concretions are formed around decaying organic matter, very rapidly, within several years, under highly constrained conditions, even when no skeletal forms occur inside. Recently, Yoshida *et al*.^3^ also found that spherical carbonate (calcite) concretions in the Miocene-age Yatsuo Group, in southern Toyama Pref., central Japan (Fig. 1), formed around the mouths of tusk-shells very rapidly over a period of weeks to months post-mortem. Owing to the very rapid formation of the concretions in the Yatsuo Group we were able to determine their ages using strontium (Sr) isotopic stratigraphy^12,13^. We propose that the method can be used generally to determine the ages of isolated carbonate concretions in marine sediments even when there are no index fossils.

The studied tusk-shell concretions occur in the upper part of the Kurosedani Formation of the Yatsuo Group and range in size from 1.5 to 3.7 cm (Fig. 1a,b)^3,4^. The upper part of the Kurosedani Formation consists of clayey sedimentary rocks that have been determined to be of lower to middle Miocene age (17 to 15 Ma) using diatom biostratigraphy and magnetostratigraphy^14–17^. Pyroclastic rocks in the Iwaine Formation, which is below the Kurosedani Formation, have a K-Ar age of 16.4 ± 0.9 Ma^14,18^.

The total thickness of the Kurosedani Formation is ca. 500 m and tusk-shell concretions are observed only in a clayey layer (about 20 metres thick) in the middle to lower-upper level of the formation. In outcrops the tusk-shells (*Fissidentalium* spp.)^15^ are seen to lie almost horizontally in the compacted clayey matrix, nearly parallel to the sedimentary layers. However, the fine layers around the concretions are bent due to compaction after concretion formation, suggesting the early formation of concretions, before consolidation of the sediment^3,4^. Isolated carbonate concretions without shell fossils inside are also found commonly in the Kurosedani Formation, with sizes varying from 2 to 7 cm.

The Yatsuo tusk-shell concretions were investigated by the following methods: rock thin-section examination by optical microscopy to determine the overall structures of the concretions and surrounding matrices; subsequently, Scanning Electron Microscope (SEM) determination of detailed matrix textures and pore geometries relevant to mass transport; followed by chemical analysis using X-ray fluorescence analyzers SXAM and XRF; and finally Sr isotopic measurements for age determination. Detailed analysis methods are given in the METHOD SUMMARY.

All tusk-shell concretions have sharp boundaries that clearly divide them from the surrounding matrices (Fig. 1), allowing their spherical shapes to be seen readily at outcrop. Optical microscopic observations of thin-section show that the tusk-shells in the concretions are well preserved as aragonite, without alteration. The micro-pores of the concretions’ matrices are tightly filled by micritic calcite. In contrast, almost no calcite fillings are observed in the surrounding host rock matrices^3^.

Major and trace element concentrations in the spherical concretions and surrounding matrices are shown in Table 1. The concretions and matrices have clearly different concentrations of Si, Al, Fe, Mn, Ca, Na, K and P, among major elements, and Ni, Cu, Zn, Ga, Rb, Sr, Y, Zr and Pb, among trace elements. Major elements except for Ca and P are mostly located in detrital grains in the concretions and matrices. All concretions have high concentrations of CaO, up to ca. 50 wt%, which are as much as 20 times higher than the concentrations in the surrounding rock matrices. Si and Al, which originate in detrital grains such as quartz and feldspar that are incorporated within the concretion, are lower than those of the surrounding matrices, reflecting the relatively high abundances of CaCO_3_ in the concretion. Among the trace elements, Sr and Y are remarkably concentrated in the concretions. In particular, Sr has been accumulated from the marine water and attains concentrations up to 950 ppm in the concretions, two to three times higher than in the surrounding rock (Table 1). It is considered that Sr and Y were accumulated from marine porewater during the concretion formation, although the Y concentration process is still unclear. Other trace elements have lower concentrations compared to the surrounding matrices.

**Table 1:** Major and trace element concentrations in concretions and buried matrices.

	Concretion part						Matrix part		
	111401	111403	111404	111405	111406	111409	111407	111408	111410
	(01^*^)	(02^*^)	(03^*^)	(04^*^)	(05^*^)	(−)	(06^*^)	(07^*^)	(08^*^)
SiO_2_	28.79	34.03	31.64	28.05	33.83	35.91	62.54	62.14	60.43
TiO_2_	0.25	0.32	0.29	0.24	0.32	0.34	0.92	0.90	0.90
Al_2_O_3_	6.68	8.15	7.43	6.46	8.08	8.73	17.79	17.51	17.14
Fe_2_O_3_	2.21	2.55	2.48	2.15	2.49	2.60	6.90	7.36	6.95
MnO	0.63	0.42	0.43	0.69	0.44	0.43	0.03	0.03	0.03
MgO	2.93	3.01	3.08	2.85	2.98	2.78	2.43	2.42	2.34
CaO	51.67	45.71	48.34	52.12	46.01	43.00	3.99	3.84	4.04
Na_2_O	0.70	0.90	0.81	0.69	0.90	1.02	1.86	1.92	1.95
K_2_O	0.67	0.89	0.80	0.64	0.89	1.05	1.79	1.78	1.75
P_2_O_5_	2.39	1.08	1.15	2.96	1.07	0.97	0.13	0.13	0.13
Total (wt%)	96.91	97.07	96.45	96.84	97.00	96.82	98.38	98.03	95.65
Cr	0.1	7.6	7.5	tr.	6.7	14	54	56	52
Co	5.3	5.9	5.7	4.7	5.4	6.5	14	13	14
Ni	tr.	tr.	tr.	tr.	tr.	6.1	24	25	25
Cu	tr.	5.2	0.7	tr.	2.1	2.0	26	24	25
Zn	25	34	30	23	33	43	109	112	116
Ga	2.1	4.3	3.3	3.0	3.7	3.7	19	20	19
Rb	14	19	17	14	19	24	70	71	69
Sr	880	880	900	850	890	950	370	350	370
Y	79	83	74	89	85	64	24	25	26
Zr	67	86	80	63	84	88	200	210	200
Nb	6.6	6.7	6.3	6.7	7.0	6.3	11	11	11
Pb	7.1	8.5	10.4	9.1	8.7	8.1	39	35	38
Th (ppm)	7.3	7.3	8.5	7.3	8.3	8.5	10	10	9.0
Ig.Loss (wt%)	32.34	30.63	31.51	32.62	30.52	29.27	12.12	12.30	12.05

Major and trace element concentrations in concretions and buried matrices determined by XRF. Major elements are expressed in % and trace ones are in ppm. Values of Total (wt%) are the total percentage of major elements in the sample after loss on ignition. Data for major element compositions are partly from Yoshida *et al*.^3^.

tr.: trace (below detection limit).

^*^Sample number shown in major elements are referred from Supplementary Table 3 of Yoshida *et al*.^3^.

A Yatsuo tusk-shell concretion, as distinguished geochemically by high concentrations of Ca and Sr analysed by SXAM, is shown in Fig. 1c,d. The Ca and Sr concentration profiles across all concretions were found to vary little, except in the central part of the concretion which presumably coincides with the region once occupied by soft tissues^3,4^. The profiles of Ca and Sr across the concretions’ rims also both decrease rapidly towards the surrounding sedimentary matrices (Fig. 1c,d). Such Sr accumulation is well known, particularly in carbonate-rich marine sediments^19^. Many carbonate-rich spherical concretions observed in marine sediments commonly accumulate Sr up to several hundreds of ppm^20^. If the Sr was co-precipitated during concretion formation, the ages determined from Sr isotopic ratios can be generally used to indicate the sedimentation ages as well as the ages of the concretions.

The ^87^Sr/^86^Sr ratios of calcite in the tusk-shell concretions in the Kurosedani Formation vary little and lie within 0.70865~0.70867, as shown in Table 2. The values are similar to those of tusk-shell aragonite, which are in the range 0.70865~0.70868. In addition, the isolated carbonate concretions without shell fossils have ^87^Sr/^86^Sr ratios of 0.70865~0.70868, which are also well consistent with the tusk-shell aragonite values. The calcite in the matrices has a wider ^87^Sr/^86^Sr range (0.70863~0.70912) than those in the tusk-shell concretions and tusk-shells. On the other hand, ^87^Sr/^86^Sr values of detrital grains of silicate minerals from the concretion and surrounding matrices lie in the range 0.70741~0.70768 (Table 3), which is clearly different from the range of values given by carbonate in the concretions and tusk-shells. We consider that the calcites in the concretions are unlikely to contain significant Sr of terrigenous (detrital) origin. This conclusion follows from the fact that if calcite in the concretions contains some Sr originating in terrigenous material, with a lower ^87^Sr/^86^Sr value of around 0.7075, the Sr isotopic ratios of the calcite will be lower than the seawater values at the formation time of the concretions and will indicate an older age as described later. However, all of the Sr isotope ages are consistent with the depositional age of the sedimentary layer as estimated from palaeontological information.

**Table 2:** Sr isotopic ratios and the corresponding numerical ages of calcite and aragonite fractions in the carbonate concretions.

Sample	fraction	^87^Sr/^86^Sr_measured_^*,a^	^87^Sr/^86^Sr_corrected_^*,b^	age (Ma)^*,c^
*tusk-shell concretion*				
111401	calcite	0.708664 +/− 0.000013	0.708651	
111403	calcite	0.708662 +/− 0.000013	0.708648	
111404	calcite	0.708670 +/− 0.000014	0.708657	
111405	calcite	0.708678 +/− 0.000011	0.708665	
111406	calcite	0.708685 +/− 0.000013	0.708671	
111409	calcite	0.708664 +/− 0.000014	0.708651	
*average*			0.708657 +/− 0.000009	17.08 (+0.27, −0.28)
*concretion without fossil*				
0708-01	calcite	0.708670 +/− 0.000011	0.708657	
0708-02	calcite	0.708657 +/− 0.000014	0.708645	
0708-03	calcite	0.708694 +/− 0.000013	0.708682	
0708-04	calcite	0.708689 +/− 0.000011	0.708676	
0708-05	calcite	0.708676 +/− 0.000011	0.708664	
*average*			0.708665 +/− 0.000014	16.95 (+0.36, −0.37)
*average of concretions with tusk shell and without fossil*			0.708661 +/− 0.000009	17.02 (+0.27, −0.27)
*tusk-shell*				
a1	aragonite	0.708694 +/− 0.000014	0.708680	
1996-01	aragonite	0.708667 +/− 0.000014	0.708654	
2014-01	aragonite	0.708682 +/− 0.000013	0.708669	
2015-01	aragonite	0.708687 +/− 0.000013	0.708675	
*average*			0.708670 +/− 0.000012	16.86 (+0.34, −0.34)
*matrix*				
111407	calcite	0.709135 +/− 0.000013	0.709121	
111408	calcite	0.708639 +/− 0.000014	0.708626	
111410	calcite	0.708706 +/− 0.000011	0.708693	

Strontium isotopic ratios, ^87^Sr/^86^Sr, of calcite or aragonite fractions from the tusk-shell concretions, concretions without fossil, host rock matrices and tusk-shells from the Yatsuo Group are shown. Based on the Sr isotopic stratigraphy, the numerical ages for the tusk-shell concretions, concretions without fossils, and tusk-shells are determined to be 17.08 (+0.27, −0.28) Ma, 16.95 (+0.36, −0.37) Ma and 16.86 ± 0.34 Ma and are fairly consistent with each other. An averaged age of concretions with tusk-shell and without fossil is 17.02 ± 0.27 Ma.

^*,a^The errors are 2 SE level on single measurements.

^*,b^The repeated analysis of NIST-SRM987 during this study gives the value of 0.710261 ± 0.000005 (2 SE, *n* = 14). ^87^Sr/^86^Sr ratio is normalized to NIST Standard Reference Material (SRM) 987 value (=0.710248). Averaged ^87^Sr/^86^Sr ratios for calcite fractions in concretions with tusk-shell, those without fossils, both of the calcites, and aragonite fractions of tusk-shells are presented with 2 SE. Each error of the ^87^Sr/^86^Sr ratio includes both error for repeated analysis of the samples and that of NIST-SRM987, that is error propagation. See “METHOD SUMMARY” in detail.

^*,c^Ages are determined by the method of McArthur *et al*.^13^. The age error for each calcite/aragonite sample is based on 2 SE for the repeated analysis of multiple samples and that of NIST-SRM987.

**Table 3:** Sr isotopic ratios of silicate fractions of carbonate concretions.

Sample	fraction	^87^Sr/^86^Sr_measured_^*,a^	^87^Sr/^86^Sr_corrected_^*,b^
*tusk-shell concretion*			
111401	silicate	0.707611 +/− 0.000011	0.707598
111403	silicate	0.707640 +/− 0.000013	0.707627
111404	silicate	0.707683 +/− 0.000014	0.707670
111405	silicate	0.707673 +/− 0.000014	0.707659
111406	silicate	0.707695 +/− 0.000013	0.707681
111409	silicate	0.707621 +/− 0.000013	0.707608
*matrix*			
111407	silicate	0.707601 +/− 0.000011	0.707588
111408	silicate	0.707448 +/− 0.000011	0.707434
111410	silicate	0.707426 +/− 0.000014	0.707413

Sr isotopic ratios of the silicate fractions from the concretions and surrounding matrices are clearly different from the range of values given by the calcite/aragonite fractions.

^*,a^The errors are 2 SE level on single measurements.

^87^Sr/^86^Sr ratio is normalized to NIST Standard Reference Material (SRM) 987 value (=0.710248). ^*,b^The value of repeated analysis of NIST-SRM987 during this study was 0.710261 ± 0.000005 (2 SE, *n* = 14).

Yoshida *et al*.^3,4^ revealed that tusk-shell concretions were formed very rapidly, post-mortem, around the mouths of tusk-shells. The concretions formed under highly constrained conditions by reactions between the decay products of organic matter and Ca^2+^ from the marine water. The similar Sr isotopic ratios obtained in the present study from the concretions (0.70865~0.70868) and the tusk-shells themselves (0.70865~0.70868) are consistent with this model. These similar ratios indicate that the tusk-shells and the surrounding concretions both accumulated Sr from the same marine water. If the concretions had formed much later, after sedimentation, the Sr would have accumulated from water with a different Sr isotopic ratio to that of the marine water in which the tusk-shells grew, in which case the Sr isotopic ratios of the concretions and tusk-shells would have been different.

**Figure 2:**
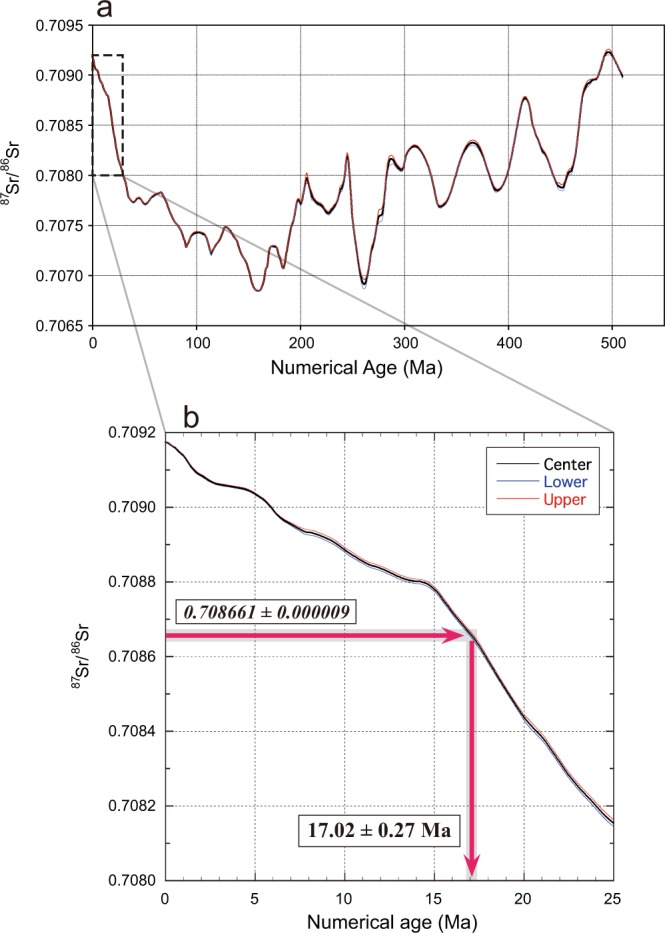
Sr isotopic stratigraphy and numerical age determination by concretions. (**a**) Numerical age determined by Sr stratigraphy based on the well-known ^87^Sr/^86^Sr variations of seawater during the Paleogene to the present^12,13^ with the accuracy within ±0.1~0.2 Ma. (**b**) The age of the tusk-shell concretions and concretions without fossils from Yatsuo Group lie within a narrow range of 17.02 ± 0.27 Ma based on the Sr isotopic stratigraphy. The centre, lower and upper lines (black, blue and red) show a best-fit line, lower-age and upper-age limits on the Sr-isotope curve^13^, respectively.

The ^87^Sr/^86^Sr variations of marine water from the Cambrian to the present, based on Sr isotopic stratigraphy, have been well established using Sr isotopic data and the fossil record from marine sediments worldwide (Fig. 2)^12,21^. An accurate Sr isotopic timescale has been determined by correlating Sr isotopic variations with stratigraphic ages based on other evidence^12^. The Sr isotopic curve is widely accepted for determining the ages of carbonate rich fossils and calcareous marine sediments^12,21^. In particular, the Sr stratigraphy from the Paleogene to the present has been well analysed and ages are accurate to within ±0.1~0.2 Ma^12,21^ (Fig. 2). Based on this Sr isotope stratigraphy and the measured ^87^Sr/^86^Sr values, the age of the tusk-shell concretions lies within a narrow range of 17.08 (+0.27, −0.28) Ma (Fig. 2 and Table 2). The age determined from the ^87^Sr/^86^Sr of the tusk-shells themselves (16.86 ± 0.34 Ma) is almost the same (Table 2). Such a high coincidence of the numerical ages between the concretions and the tusk-shells implies that all the concretions formed sufficiently rapidly to accumulate Sr from the marine water present at the time the tusk-shell lived. Both the Sr isotopic ratios of concretions and the tusk-shells consistently indicate that the concretions were formed within a very short time after the decomposition of the life forms. All evidence suggests that Sr isotopes were rapidly fixed during concretion formation and that the isotopic ratios have not changed thereafter by further calcite precipitation within the spherical concretions.

The estimated sedimentation age of the Yatsuo Group based on microfossil biostratigraphy and magnetostratigraphy^14–17^ has a relatively wide range of 17 to 15 Ma, because of the limited accuracy of fossil time ranges and magnetostratigraphical intervals^15^. On the other hand, Sr isotopic data shown in this study have a much higher resolution than the fossil age estimations. Our study also indicates that isolated carbonate concretions without index fossils inside, in marine sediments, can be used directly to determine Sr isotopic numerical ages with high accuracy. The age determined from the ^87^Sr/^86^Sr of concretions without fossils, 16.95 (+0.36, −0.37) Ma, is almost the same as that of the tusk-shell concretions (Table 2). Strontium isotopic stratigraphy using ^87^Sr/^86^Sr ratios of all concretions with tusk-shell and without fossils indicates an age of 17.02 ± 0.27 Ma.

In summary, Sr isotopic data from all concretions and the tusk-shells show that the concretions were formed sufficiently rapidly to preserve the isotopic ratio of marine water Sr at the time of sedimentation. These results are consistent with our recent examination of several kinds of concretions from marine sediments of different ages. This earlier study revealed that spherical carbonate concretions without fossils inside were formed rapidly after sedimentation by reactions involving the decay products of organic matter once present inside them^4^. In marine clayey sediments, isolated spherical carbonate concretions are commonly observed but do not always contain index fossils that can be used to estimate sedimentation ages. Hence, concretions without fossils inside have never previously been used for age determination. However numerical ages derived from the ^87^Sr/^86^Sr isotopic ratios of the concretions examined here indicate that the methodology can be applied to estimate the ages of all kinds of spherical concretions in marine sediments.

### Method Summary

Thin-sections were prepared from Epoxy resin-impregnated rock samples by cutting the samples across the centres of tusk-shell concretions. The sections were orientated to characterize the textures preserved inside the concretions and tusk-shells. The rock thin-sections were also used to determine the major and Sr distributions in and around the concretions by SXAM (XGT-2000V Horiba Japan) at the Department of Education, Gifu University, Gifu, Japan. SXAM intensity maps of concretions were reduced to one-dimensional element profiles in a direction perpendicular to the concentric ring patterns identified in elemental maps of the concretions, using the lamination trace technique^22^. Each measurement was made with a high-intensity continuous X-ray beam (Rh anode 50 kV 1 mA) with 100 μm in diameter. The beam was focused with a guide tube perpendicular to the surface of a sample, which was located on a PC-controllable X-Y stage. X-ray fluorescence from the sample surface was analyzed with the hp-Si detector of an energy-dispersion spectrometer. The results show semi-quantitatively the two-dimensional distribution of all elements across the whole surface of each sample.

Samples were carefully collected and prepared from each concretion and the surrounding matrix. XRF analyses were undertaken to measure major and minor element contents quantitatively using a Rigaku ZSX Primus II WD-XRF spectrometer equipped with a Rh X-ray tube at the Graduate School of Environmental Studies, Nagoya University. Glass beads were prepared by mixing a portion of each sample, which was ignited at 950 °C to decompose carbonates, with anhydrous lithium tetraborate flux and then fusing. Measurements were calibrated with mixed samples of rock reference sample JLs-1 with sedimentary and igneous ones issued by the Geological Survey of Japan (GSJ: Geochemical Reference Sample Data Base, https://gbank.gsj.jp/geostandards/welcome.html). Analytical uncertainties were estimated to be 1 to 2% for SiO_2_ and Al_2_O_3_, 5% for the rest of the major elements, and 10% for trace elements.

Samples of tusk-shell, surrounding concretion and rock matrix from outside the concretion, were used for Sr isotope analysis. Each sample, weighing 10–50 mg, was dissolved in 2 ml of 10% acetic acid at room temperature for 3 h. Then the sample was centrifuged for 15 min at 3500 rpm, and the residue was removed. The dissolved fractions from the tusk-shells, concretions and rock matrices are mostly calcite or aragonite. The strontium in the dissolved fraction was purified using cation-exchange chromatography (BioRad AG50W- X8, 200–400 mesh) with a 2.4 M HCl eluent. The residue was dried at 85 °C for 12 h and weighed, and the residue weight percentage was determined. The residual fractions from the concretion and surrounding rock matrices are mostly detrital silicate. Dissolution of the tusk-shell leaves hardly any residue. The residual silicate fraction was decomposed by HF + HClO_4_, and then strontium was separated from the silicate fraction by cation-exchange chromatography.

Strontium isotopic ratios, ^87^Sr/^86^Sr, were measured by a VG Sector 54-30 thermal-ionization mass spectrometer and normalized to a ^86^Sr/^88^Sr value of 0.1194. The mean value of the NIST-SRM 987 standard run with the samples was ^87^Sr/^86^Sr = 0.710261 ± 0.000005 (2 SE, *n* = 14). The ^87^Sr/^86^Sr values of the samples have been corrected for inter-laboratory bias by adjusting the mean value of the NIST-SRM987 standard run with the samples to the value of 0.710248 stated by McArthur *et al*.^13^.

Ages are determined by the method of McArthur *et al*.^13^. The age error for each calcite/aragonite sample is based on 2 SE for the repeated analysis of multiple samples and that of NIST-SRM987. For example, in the case of calcite fractions for tusk-shell concretions with a value of ^87^Sr/^86^Sr = 0.708657 ± 0.000009, the averaged value of 0.708657 gives a mean age of 17.08 Ma, the value of 0.708666 (average + 2 SE) gives a lower age limit of 16.80 (=17.08 − 0.28) Ma, and the value of 0.708648 (average − 2 SE) gives an upper age limit of 17.35 (=17.08 + 0.27) Ma. Averaged ages for calcite fractions of the tusk-shell concretions, concretions without fossils, and for aragonite fractions of tusk-shells, are simple mean values with 2 SE.
