# Publisher Correction: ^87^Sr/^86^Sr age determination by rapidly formed spherical carbonate concretions

**DOI:** 10.1038/s41598-019-40680-w

**Published:** 2019-05-07

**Authors:** Hidekazu Yoshida, Yoshihiro Asahara, Koshi Yamamoto, Nagayoshi Katsuta, Masayo Minami, Richard Metcalfe

**Affiliations:** 1 Material Research Section, Nagoya University, University Museum, Chikusa Nagoya, Japan; 2 Graduate School of Environmental Studies, Nagoya University, Chikusa Nagoya, Japan; 3 Faculty of Education, Gifu University, Yanagido Gifu, Japan; 4 Institute for Space-Earth Environmental Research, Nagoya University, Chikusa Nagoya, Japan; 5 Quintessa UK, Newtown Road, Henley-on-Thames, Oxfordshire UK

Correction to: *Scientific Reports* 10.1038/s41598-019-38593-9, published online 30 January 2019

The publication date was omitted from the original PDF version of this Article. This error has now been corrected in the PDF version; the HTML version was correct from the time of publication.

